# A Costly Misdiagnosis: Reframing AI as Augmented Intelligence in Medicine and Medical Publishing

**DOI:** 10.1111/jgs.70495

**Published:** 2026-05-05

**Authors:** Richard G. Stefanacci

**Affiliations:** ^1^ Thomas Jefferson University Jefferson College of Population Health Philadelphia Pennsylvania USA

**Keywords:** artificial intelligence disclosure, augmented intelligence, author accountability

Medicine relies on accurate diagnosis. It is therefore worth noting, with some irony, that the medical community has collectively misdiagnosed artificial intelligence (AI). The symptoms are real—fabricated references, unverifiable authorship, patient safety concerns, eroding peer review standards, fears of deprofessionalization—but the diagnosis drawn from those symptoms has been wrong: that AI itself is the pathogen, and that quarantine is the cure. The cost of that misdiagnosis is accumulating. Clinicians are avoiding tools that could improve patient care. Researchers are concealing responsible AI use out of professional fear. And a reflexive bias has taken hold: the assumption that anything touched by AI is somehow lesser, regardless of the expertise, effort, and judgment invested in its production. That bias is not rigor. It is misdiagnosis.

I have a personal stake in this argument. In 2023, I published a Letter to the Editor in this journal addressing misconceptions about AI in geriatric medicine [1]. That letter was generated almost entirely by ChatGPT from a single carefully crafted prompt. I changed exactly one word—correcting the AI's misidentification of a “Commentary” as a “Review.” The piece was peer‐reviewed, accepted, and published. My contribution was the clinical and editorial expertise embedded in the prompt and the judgment applied in review. That is not a compromise of scholarship. That is scholarship. The appropriate term for what I practiced is not “artificial intelligence” but Augmented Intelligence—AI directed and validated by an expert who retains full accountability for every element of the result.

The current disclosure debate misses this distinction entirely. Journals and publishers have constructed a binary requirement—“AI was used” or it was not—that communicates nothing meaningful about the nature or extent of the human contribution. In practice, expert AI use is not a transaction but a process: an initial framework developed independently, a first draft generated and found inadequate, the prompt redirected with additional clinical and editorial direction, the second output restructured by hand, a third platform consulted to check a factual claim, the argument refined across multiple iterations and multiple AI systems. By the time a manuscript is complete, no checkbox can describe what occurred. Worse, the disclosure requirement creates a perverse equivalence between the expert who drove that entire process and the user who submitted an unedited first output. Both “used AI.” The policy cannot tell them apart—and the resulting stigma is causing responsible users to conceal their work rather than stand behind it.

The calculator offers the instructive precedent. No journal requires disclosure that statistical analyses were performed by software rather than computed by hand. No reviewer discounts a study because its regression model was machine‐assisted. What has always mattered is whether the methodology was sound, the analysis correctly applied, and the interpretation valid—and whether the author can defend every element of the work. That standard already addresses every legitimate concern underlying the AI disclosure debate. Author accountability is not a supplement to disclosure. It is the complete answer to it.

Augmented Intelligence, properly understood, rests on three irreplaceable human contributions. The first is the question: the expert prompt that encodes years of domain knowledge, clinical context, and precision of purpose. A vague prompt yields a generic answer; an expert prompt yields something genuinely useful. The second is the guidance: the iterative steering across many versions and often multiple AI platforms, each directed by the expert's judgment about what the work still needs. This process is cognitively demanding—it requires holding the correct answer in mind in order to evaluate every generated version against it. The third is the verification: the expert validation that catches what AI gets wrong. The single‐word correction in my 2023 JAGS letter was not incidental; it was clinical and editorial knowledge catching an error that would have mischaracterized a colleague's work in print. The author who signs AI‐assisted work stakes their professional reputation on every element of it. That accountability is real, irreducible, and entirely unaffected by which tools produced the draft.

There is also a generational dimension worth naming. Those who trained before AI existed—who memorized pharmacology, built diagnostic reasoning without algorithmic assistance, and accumulated clinical judgment over decades of direct patient care—carry a critical advantage: they know what a right answer looks like. They have the internalized foundation to recognize when AI is clinically sound and when it is confidently wrong. This generation did not need to distrust AI because they could not evaluate it. They built the referent against which AI output is measured. That is not obsolescence. It is the most important qualification for using this tool responsibly.

Medicine and its journals should retire the binary disclosure requirement and replace it with the standard that has always governed scholarly work: author accountability. The author is responsible for the accuracy of every claim, the validity of every citation, and the soundness of every recommendation—regardless of what tools assisted in production. A work that is accurate, methodologically sound, and clinically significant is not diminished because AI assisted in drafting it. The question editors and reviewers should ask is not “Did you use AI?” but “Can you defend the accuracy and integrity of everything you have signed?” That question already existed. It already applies. And applying it consistently—without the stigma attached to a disclosure label—is the correct treatment for a costly misdiagnosis that medicine can no longer afford.

*Medicine and its journals should retire the binary disclosure requirement and replace it with the standard that has always governed scholarly work: author accountability*.

## Author Contributions

Richard G. Stefanacci conceived, directed, and verified all content. AI tools were used as instruments of that process across multiple platforms and iterations, consistent with the Augmented Intelligence framework described herein.

## Funding

The author has nothing to report.

## Conflicts of Interest

The author declares no conflicts of interest.
